# Supplementary material for: The Impacts of COVID-19 on Healthcare Quality in Tertiary Medical Centers—A Retrospective Study on Data from Taiwan Clinical Performance Indicators System
Source: Int J Environ Res Public Health. 2022 Feb 17;19(4):2278. doi: 10.3390/ijerph19042278 (PMC8871675; doi:10.3390/ijerph19042278)
Supplement: Supplementary file 1 [file ijerph-19-02278-s001.zip › ijerph-1569840-supplementary.pdf]

Supplementary Table S1. Definition of quality indicators (QI)

QI Code	QI name	Numerator	Denominator
AMI-07	Proportion of STEMI patients receiving PPCI within 90 minutes after arrival of ER	No. of STEMI patients receiving PPCI within 90 minutes after arrival of ER	No. of STEMI patients receiving PPCI after arrival of ER
ED-Leav-01	Proportion of patients leaving ER without complete treatment	No. of patients without complete treatment after registration at ER	Total No. of patients' registration at ER
ED-UnR-02	Unscheduled returns within 72 hours for the emergency patients	No. of unscheduled returns within 72 hours for the emergency patients	Total No. for the emergency patients
Hosp-Mort-02	In-hospital mortality rate (excluding critical DAMA)	No. of mortality patients	Total No. of discharged patients
Hosp-UnR-01	Unscheduled returns within 14 days for the hospitalized patients	No. of unscheduled returns within 14days for the hospitalized patients	Total No. for the hospitalized patients
Sc-Mort-02	Mortality within 48 hours for the surgical patients (excluding critical DAMA)	No. of mortality within 48 hours for the surgical patients (excluding critical DAMA)	Total No. of surgical patients (in-hospital)
Sc-UnR-01	Unscheduled returns to the OR for the surgical patients during hospitalization	No. of unscheduled returns to the OR for the surgical patients during hospitalization	Total No. of surgical patients (in-hospital)
STK-03	Proportion of acute ischemic stroke patients receiving IV-tPA	No. of acute ischemic stroke patients receiving IV-tPA	Total No. of acute ischemic stroke patients

Abbreviation: QI: Quality indicator; STEMI: ST-elevation myocardial infarction; PPCI: Primary percutaneous coronary intervention; OR: Operation room; ER: Emergency room; No: Number; DAMA: Discharge against medical advice;
